# The Yin and Yang of the oxytocin and stress systems: opposites, yet interdependent and intertwined determinants of lifelong health trajectories

**DOI:** 10.3389/fendo.2024.1272270

**Published:** 2024-04-16

**Authors:** Kerstin Uvnäs-Moberg, Mechthild M. Gross, Jean Calleja-Agius, Jonathan D. Turner

**Affiliations:** ^1^ Department of Animal Environment and Health, Section of Anthrozoology and Applied Ethology, Swedish University of Agricultural Sciences, Skara, Sweden; ^2^ Midwifery Research and Education Unit, Hannover Medical School, Hannover, Germany; ^3^ Department of Anatomy, Faculty of Medicine and Surgery, University of Malta, Msida, Malta; ^4^ Immune Endocrine Epigenetics Research Group, Department of Infection and Immunity, Luxembourg Institute of Health, Esch sur Alzette, Luxembourg

**Keywords:** stress, HPA axis, oxytocin system, early life adversity, developmental origins of health and disease, somatosensory nerves, limbic system

## Abstract

During parturition and the immediate post-partum period there are two opposite, yet interdependent and intertwined systems that are highly active and play a role in determining lifelong health and behaviour in both the mother and her infant: the stress and the anti-stress (oxytocin) system. Before attempting to understand how the environment around birth determines long-term health trajectories, it is essential to understand how these two systems operate and how they interact. Here, we discuss together the hormonal and neuronal arms of both the hypothalamic-pituitary-adrenal (HPA) axis and the oxytocinergic systems and how they interact. Although the HPA axis and glucocorticoid stress axis are well studied, the role of oxytocin as an extremely powerful anti-stress hormone deserves more attention. It is clear that these anti-stress effects depend on oxytocinergic nerves emanating from the supraoptic nucleus (SON) and paraventricular nucleus (PVN), and project to multiple sites at which the stress system is regulated. These, include projections to corticotropin releasing hormone (CRH) neurons within the PVN, to the anterior pituitary, to areas involved in sympathetic and parasympathetic nervous control, to NA neurons in the locus coeruleus (LC), and to CRH neurons in the amygdala. In the context of the interaction between the HPA axis and the oxytocin system birth is a particularly interesting period as, for both the mother and the infant, both systems are very strongly activated within the same narrow time window. Data suggest that the HPA axis and the oxytocin system appear to interact in this early-life period, with effects lasting many years. If mother-child skin-to-skin contact occurs almost immediately postpartum, the effects of the anti-stress (oxytocin) system become more prominent, moderating lifelong health trajectories. There is clear evidence that HPA axis activity during this time is dependent on the balance between the HPA axis and the oxytocin system, the latter being reinforced by specific somatosensory inputs, and this has long-term consequences for stress reactivity.

## 1 Introduction

Over the last three decades it has become clear that lifelong health trajectories are determined between conception and age 2, often referred to as the “first 1000 days of life” (1–3). David Barker made the seminal observation that birthweight was linked to the development of cardiovascular disease later in life (4, 5). Over time, this has been expanded and early-life conditions have been shown to play a large part in programming adult-onset illnesses such as cardiovascular disease (6), asthma (7), cancer (8) and mental disorders including anxiety and depression later in life, and this phenotype has been successfully reproduced in rodent models (9–13). This has now been refined into the Barker hypothesis or the “developmental origins of health and disease” where these first 1000 days are considered to be an especially sensitive developmental window, during which almost all biological systems are put in place and their plasticity allows them to be fine-tuned to the immediate environment (14). A lot of effort has been expended looking at the long-term effect of negative psychosocial and physical situations such as parental mental health, low socio-economic status, and paradigms such as abandonment and/or institutionalization. However, the natural process of childbirth is the first time at which the infant is usually exposed to a major stressor – i.e. birth. At the same time, they are usually exposed to high levels of oxytocin. Differing modes of birth and clinical practices are associated with this hormonal balance and will have many similar effects to those caused by negative psychosocial and physical situations.

During parturition and the immediate post-partum period there are two opposite, yet interdependent and intertwined systems that are highly active and play a role in determining lifelong health and behaviour in both the mother and her infant: the stress system and the anti-stress (oxytocin) (15). Before attempting to understand how the environment around birth determines long-term health trajectories it is essential to understand how these two systems operate and how they interact. The structure, and the effect patterns, of the stress system, including the hypothalamic-pituitary-adrenal (HPA)- axis and the sympathetic nervous system (SNS), are well known, whereas the knowledge of the structure and effects of the oxytocinergic system is not so widespread. Oxytocin from peripheral and central sources, together with both peripheral and central effects of oxytocin makes up the oxytocinergic system (16). Oxytocin exerts many important psychological and physiological effects, such as stimulation of social interaction, bonding, increased well-being and decreased fear, stress and pain. Oxytocin also has an important anti-stress facet; these effects are exerted at multiple sites in the brain and include direct inhibitory actions on the HPA axis and the SNS as well as more indirect effects e.g. via the amygdala and the Locus Coeruleus (LC).

In this paper, we will summarize the structure and the functional organization of the stress and oxytocin systems. The basics functionality of the oxytocin system with pre-peptide production, processing, receptor activation and interaction with the vasopressin system has been reviewed elsewhere (17) as well as the basic principle of the anti-stress effect of oxytocin In particular, we focus on the anti-stress effects of oxytocin by describing pathways and mechanisms involved in the stress-reducing effects of oxytocin. We will include both the more direct anti-stress effects of oxytocin and the mechanisms by which oxytocin can influence the activity of the stress system in a more indirect way. Further attention will be given to oxytocin release and anti-stress effects caused by sensory stimulation via the autonomic nervous system and cutaneous afferents. These effects are of importance during birth, skin-to-skin contact between parents and their newborns and in connection with breastfeeding in both mother and baby. This balance between the stress and the oxytocin systems is of utmost importance for the outcome of these processes from both a physiological as well as a psychological point of view. Understanding these the interplay between these two opposite, yet interdependent and intertwined systems will eventually allow us to harness the power of the endogenous oxytocin system to buffer noxious or toxic stress reactions to avoid long-term effects of various types of traumatic events.

## 2 The two arms of the physiological stress response

**Figure 1:**
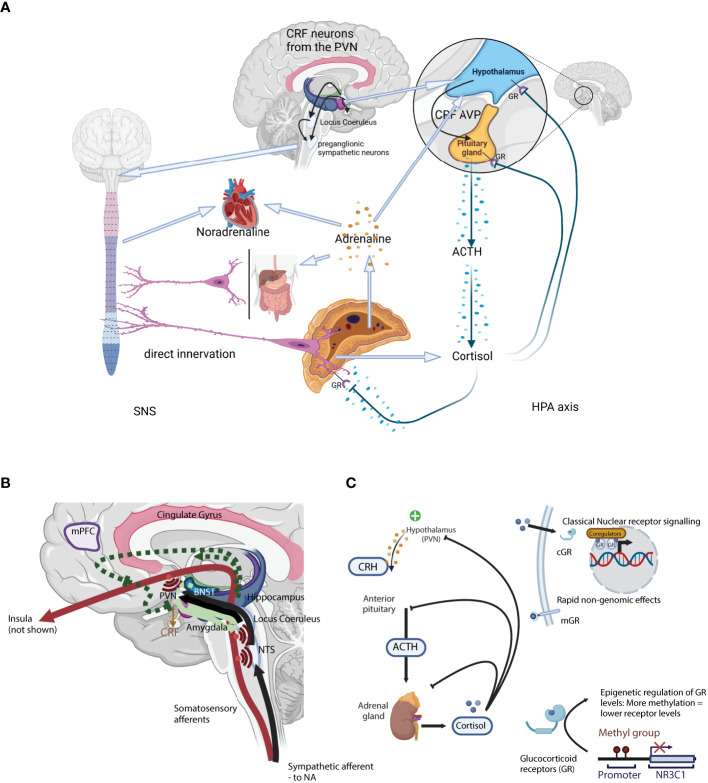
**(A)** A schematic representation of the two arms of the stress system: the sympathetic nervous system (SNS; Left) and the Hypothalamus-Pituitary-Adrenal axis (HPA axis; Right). **(B)** Neuronal control of the HPA axis from physical stressors such as blood loss, hypoglycaemia, pain, or immune challenge. Here, direct mono-synaptic ascending sensory projections or A1 and A3 noradrenergic bundles go through the brainstem, the NTS, and from there directly into the PVN (21) where they have direct synaptic contact with CRH-secreting PVN neurons. Furthermore, NTS neurons project into limbic regions such as the dorsomedial hypothalamic nucleus (DMH, modulates ANS activity), dorsal raphe (orchestrates serotoninergic activity) as well as the forebrain. In addition to the direct projections, NTS neurons projecting into the PVN also secrete neuropeptides like glucagon-like peptide 1 and NPY, that act on CRH-secreting neurons to moderate HPA axis activity. Light green arrows – CRH neurons. Solid lines: direct innervation, dotted lines: indirect actions. = Axon collaterals. **(C)** A schematic representation of the classical hormonal arm of the HPA axis. Secretion of CRH from the PVN initiates the classical HPA axis “action arm” incorporating the hormonal cascade via the intermediary adreno-corticotropic hormone (ACTH), ending with adrenal GC secretion (22, 23). GC act through specific receptors, the glucocorticoid receptor (GR) and the mineralocorticoid receptor, to provide negative feedback in the HPA axis, essential for terminating the stress response (24). Negative feedback is bi-phasic with rapid and slow phases, acting in seconds/minutes and hours/days respectively. CRH, Corticotropin-releasing hormone; ACTH, Adrenocorticotropic hormone; AVP, arginine vasopressin; GR, glucocorticoid receptor; PVN, paraventricular locus of the hypothalamus; HPA axis, hypothalamus-pituitary-adrenal axis; NTS, nucleus tractus solitarii.

The physiological response to a physical or psychosocial stressor originates in the hypothalamic paraventricular nucleus (PVN), activating both the hypothalamus—pituitary—adrenal (HPA) axis (18) and the autonomic nervous system (ANS) (19, 20) (**Figure 1A**). These two arms of the stress response act synergistically. The first step, common to the two arms of the stress response, is the secretion of corticotropin-releasing hormone (CRH; official gene symbol *CRH*, often called corticotropin-releasing factor CRF) from specific CRH-secreting neurons in the PVN. CRH release stimulates the HPA axis, while CRH-secreting neurons also directly activate the ANS *via* preganglionic neurons of the sympathetic chain as well as indirectly by projecting to brainstem noradrenergic centres such as the LC (25) (**Figure 1A**).

Although the HPA axis is often reduced to the hormonal cascade from the hypothalamus to the adrenal cortex *via* the pituitary gland (the downstream “action network”), there are a complex series of polysynaptic pathways involved in the activation and extinction of the HPA axis (the “upstream regulatory network”). CRH secretion marks the end of the latter central process, and the start of the hormonal cascade. The HPA axis is regulated at both levels, either through a complex neuronal network modulating the eventual CRH secretion from the PVN (the “upstream regulatory network”), or through a glucocorticoid receptor (GR) and GC-mediated hormonal feedback loop (**Figure 1A**).

At the same time as secreting CRH via the median eminence for further transport to the anterior pituitary, CRH-secreting neurons project to integrative stress centres in the brain stem, such as the LC (25) which in turn also activates the autonomic nervous system (ANS). Upon activation of the sympathetic arm (SNS) of the ANS, cervical preganglionic sympathetic neurons are activated, including those that project directly to chromaffin cells in the adrenal medulla from where the catecholamines adrenaline and noradrenaline are released (25). Rapid catecholamines release induces a transient increase in both blood pressure and heart rate. CRH secreting fibres also project to the dorsal motor nucleus of the vagal nerve (DMX) and the nucleus tractus solitarius (NTS), where they inhibit parasympathetic nervous function. The parasympathetic arm (PNS) of the ANS tempers and controls the SNS equilibrating the system and maintaining homeostasis. SNS and PNS activities are balanced and adapted to the external environment e.g. through selective activation of the α1- and α2-adrenoceptors in the LC (26, 27). Unlike the HPA axis, SNS activation activates a positive feedback loop, increasing PVN- CRH secretion (28).

### 2.1 Neuronal inputs into the PVN activating and controlling the stress response

The PVN integrates the many varied direct inputs such as basic circadian drive from the suprachiasmatic nucleus (SCN) and integrates them with the emotional, cognitive, physical and reflex actions and reactions to correctly adapt the physiological stress response to the environment experienced (29, 30). These can be broadly categorised into stressors that require an immediate response and act via direct ascending projections from the spinal cord and brainstem into the PVN, and slower more complex cognitive process involving multiple interconnected limbic structures such as the amygdala, hippocampus and pre-frontal cortex.

#### 2.1.1 Rapid effects

Physical stressors such as blood loss, hypoglycaemia, pain, or immune challenge that require a rapid physiological response all signal through direct mono-synaptic (ascending) sensory projections or as in the case of sympathetic afferent nerves via A1 and A3 noradrenergic bundles in the brain stem after relaying into the NTS and from there directly into the PVN (21) (**Figure 1B**). These projections have direct synaptic contact with CRH-secreting PVN neurons. Furthermore, NTS neurons project into limbic regions such as the dorsomedial hypothalamic nucleus (DMH, modulates ANS activity), dorsal raphe (orchestrates serotoninergic activity) as well as the forebrain. In addition to the direct projections, NTS neurons projecting into the PVN also secrete neuropeptides like glucagon-like peptide 1 (GLP-1) and NPY, that act on CRH-secreting neurons to moderate HPA axis activity (reviewed in (31)).

#### 2.1.2 Complex psychological and psychosocial stressors

Unlike the rapid response to physical and metabolic stressors, complex psychological and psychosocial stressors depend on polysynaptic pathways that are much more complex (31). These involve limbic structures such as the bed nucleus of the stria terminalis (BNST), together with the medial prefrontal cortex (mPFC), hippocampus, and amygdala initiating the HPA axis and ANS stress responses (**Figure 1B**) (32). CRH neurons originating in the central amygdala project to the LC where noradrenergic fibres which project to the PVN are activated (31). This is the most important pathway by which the endocrine HPA axis is activated. In contrast, some prelimbic and dorsomedial PFC projections inhibit both HPA axis and ANS activity (33).

### 2.2 Polysynaptic pathways in the upstream “regulatory” arm in the response to psychological and psychosocial stressors

#### 2.2.1 Hippocampal inputs

The hippocampus is traditionally thought to play a role in spatial cognition, episodic memory, and response inhibition. Although the response inhibition role has lost favour, the hippocampus is involved in the analysis of new stimuli as well as detecting new places and events and is now considered part of the approach-avoidance system linked to anxiety and the stress response (34). The hippocampus indirectly feeds ANS and HPA axis inhibitory signals into the PVN through the ventral subiculum (HPA axis) and PFC (ANS) which subsequently activate GABAergic and glutamatergic projections to the CRH-secreting PVN neurons (33). Hippocampal inhibition reduces basal GC levels, as well as shortening the duration of the HPA axis stress response, although the magnitude of the GC response appears unaffected (31, 35, 36).

#### 2.2.2 Amygdala inputs

The amygdala, together with the hippocampus plays a role in emotional processing and response to stimuli, including negative sensitivity and anxiety (37–39). Sensory information projects into the lateral amygdala and is passed to the central nucleus of the amygdala after processing. Upon stress, CRH neurons in the central nucleus amygdala are activated to induce fear signalling and via direct projections to the LC to induce activation of NA release and consequent activation of the PVN. Furthermore, the medial and basolateral amygdala have inhibitory actions, reducing HPA axis stress reactivity (31).

#### 2.2.3 Bed nucleus of the striata terminalis

The BNST integrates limbic inputs, particularly from the hippocampus and the amygdala, to the PVN and eventually the brain stem (31). Neurons in the information-outflow regulating oval nucleus of the BNST (ovBNST) secrete CRH (40, 41). These CRH-secreting neurons, through indirect projections to CRH-secreting neurons in the PVN, modulate the HPA axis and ANS stress response (31, 42). These indirect projections most probably involve synapses with periventricular glutamatergic and GABAergic neurons, although the BNST contains predominantly inhibitory GABAergic neurons, and excitatory messages most probably occur through their inhibition (31). The BNST is an important centre of HPA axis and behavioural regulation after exposure to early-life adversity (43). Early-life adversity induces long-term CRH signalling activation (43), although contrary to the human hypoactive HPA axis phenotype (44).

#### 2.2.4 Locus coeruleus

The LC - noradrenergic system (LC-NA system) is a small, dense, sub-region of the pons that projects in a bridge-like manner the spinal cord and the forebrain. The LC is central to the stress response, as well as many other physiological functions, such as pain processing, cognition, memory, and arousal (45). Furthermore, the LC is involved in the stress-anxiety circuit and stress-induced neuropsychiatric disorders, e.g. posttraumatic stress disorder (PTSD) (46).

### 2.3 The classical hormonal arm of the HPA axis

Secretion of CRH from the PVN initiates the classical HPA axis “action arm” incorporating the hormonal cascade via the intermediary adreno-corticotropic hormone (ACTH), ending with adrenal GC secretion (22, 23). GC act through specific receptors, the glucocorticoid receptor (GR) and the mineralocorticoid receptor, to provide negative feedback in the HPA axis, essential for terminating the stress response (**Figure 1C**; reviewed in (24)). Negative feedback is bi-phasic with rapid and slow phases, acting in seconds/minutes and hours/days respectively. Rapid inhibition of the hormonal cascade is through inhibition of CRH and ACTH secretion from the PVN and the pituitary gland, while the slow phase depends on the suppression or reduction of CRH and ACTH transcription (24).

In the absence of a stressor, GC levels follow a natural circadian and ultradian rhythm, peaking around waking, and a nadir at the end of the active phase. GC levels are the product of pulsatile GC secretion from the adrenal gland and the natural metabolism inactivating GC. Pulsatile GC secretion occurs at ~1 h intervals (IPI), and modulation of the pulse amplitude and the overall mass secreted during each episode determines the GC concentration profile (47–49) (**Figure 1C**). Rising GC concentrations during the secretory pulse act via the GR-mediated negative feedback loop to rapidly terminate the pulse, and after a fixed interval the subsequent pulse is triggered (50). This is then overlaid by the HPA axis response to a stressor, with additional pulses generated, although in the period immediately following the pulsatile release of cortisol there is a quiescent period in which the HPA axis cannot be activated (refractory period).

#### 2.3.1 Negative feedback regulation of the classical hormonal HPA axis

Negative feedback of the hormonal HPA axis occurs through direct GC-GR interactions, and is directly dependent on GR levels. Feedback clearly occurs at the classically cited PVN (51) and GC injection into the PVN strongly reduces CRH expression and the activity of the cascade lower down (31). Despite intensive efforts, GC feedback sites are still somewhat controversial. GC negative feedback is clearly not limited to HPA axis tissues (PVN, pituitary, adrenal gland) (31), as forebrain limbic structures e.g. hippocampus and amygdala are important in regulating both baseline HPA axis activity, HPA axis reactivity, but also negative feedback (52). The hippocampus expresses high levels of GR (51) and MR (53, 54), while the amygdala and prefrontal cortex express mainly the GR (51) that is activated at peak GC levels from either the circadian rhythm or upon HPA axis activation (55). Direct infusion of GC into the medial frontal cortex shuts down the HPA axis (56) and deleting the GR in the amygdala, hippocampus or cortex induces hyperactivity (57). The rapidity of the GC negative feedback implies non-genomic effects and albumin-bound GC induce the same effects without passing the cell membrane (58), most probably acting via cell membrane bound GR (59).

The negative feedback, based on the direct GC-GR interaction is thought to be directly dependent upon GR levels. We demonstrated that GR levels are maintained by an exquisite transcriptional mechanism, whereby the GR gene (*NR3C1*) uses a series of 9 non-coding alternative first exons with independent tissue-specific promoters that are subject to epigenetic regulation to maintain GR levels (60–64). The external environment, such as exposure to early-life stress induces differential methylation of these promoters (60, 65), especially around promoter 1F (66–70), orthologous to the region originally identified by Weaver et al. as controlling HPA axis reactivity lifelong (61, 71). Negative feedback sensitivity depends on functional GR levels, themselves dependent on either sequestration of the GR with the co-chaperone FK506 binding protein 51 (FKBP5), or the reduction of GR transcript levels themselves (72). However, CRH is not regulated in the same manner in all tissues. In the PVN, CRH expression is downregulated by GC/GR interactions, however, in the amygdala and the BNST CRH is upregulated by GC (73), suggesting that the negative feedback loop is far from understood (52).

Here, we have presented how the stress system incorporates a wide variety of structures within the brain that interpret external stimuli as either real or prospective dangers and goes on to induce the adequate physiological reaction via the SNS and the HPA axis. There are a plethora of stressors engaging diverse central networks, necessitating a fine-tuned function-based neuroanatomical process. Within the HPA axis, this occurs through the GR-GC negative feedback loop. It is interesting, because, this loop is also particularly sensitive to stimulation of the oxytocin system, and the two systems interact at this point.

#### 2.3.2 Transcriptional control of the GR links the HPA axis feedback loop to the oxytocin system

Despite the almost exclusive focus on the epigenetic control of *NR3C1* expression and GR levels over the past decade, there are more direct mechanisms. Almost 20 years ago we showed that hippocampal GR mRNA levels are dependent on oxytocin levels. Subcutaneous administration of oxytocin to rats over a period of 5 days decreased GR mRNA levels in the principal hippocampal regions (CA1, CA2, DG, and non-statistically in CA3), with the potential to alter the HPA axis feedback mechanism (74). Oxytocin signals through the oxytocin receptors (OTRs) that are widely distributed throughout the brain including all the HPA axis mediating regions: PVN, hippocampus, amygdala and frontal cortex (75, 76). However, immunohistochemistry shows that although GR and OTR are expressed in the same regions, in the hippocampus it would appear that oxytocin neurons are surrounded by GR-positive neurons, although they were rarely on the same neurons (77), although the mechanism may not be so simple, as OTR knockout mice are deficient in social behaviour, but have an intact HPA axis stress response (78), suggesting that the effects of oxytocin on the stress system is complex. Exogenous oxytocin administration decreases adrenal weight and volume (79) and reduces CRH mRNA levels in the PVN (80, 81) demonstrating the clear interaction between the two systems.

## 3 The two arms of the oxytocin system

**Figure 2:**
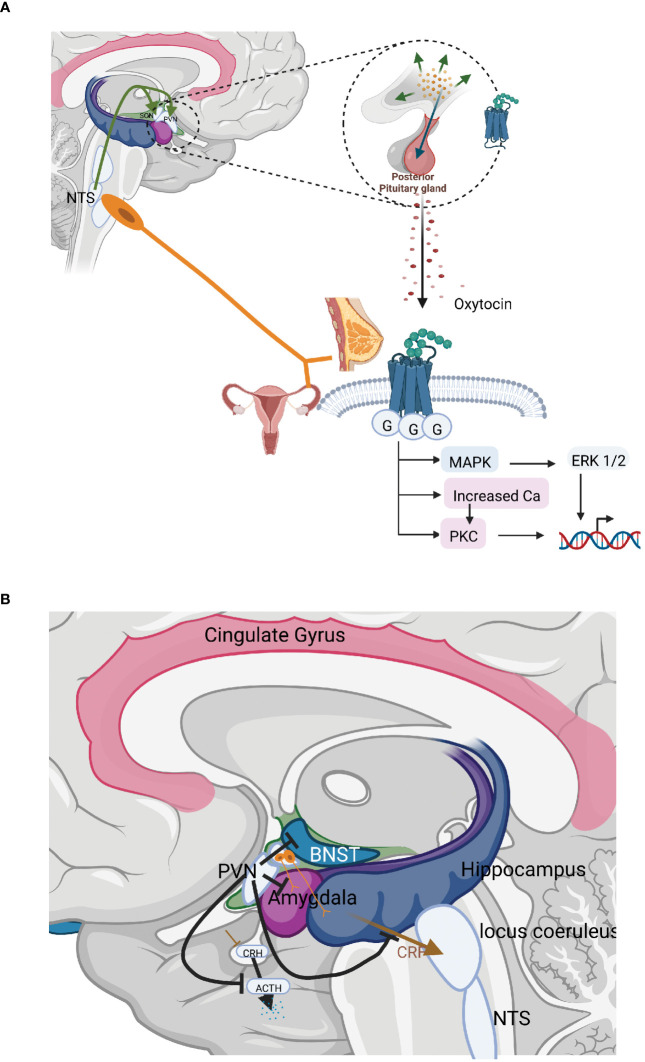
**(A)** The organisation and actions of the hormonal and neurotransmitter arms of the oxytocin system. The most well-known effects of oxytocin are stimulation of uterine contractions during birth and milk ejection in connection with breastfeeding. In these situations oxytocin is released from the anterior pituitary into the circulation and thus acts as a hormone. Oxytocin may, however, also be released as a neurotransmitter in the brain inhibiting stress levels. **(B)** Central interaction sites between the HPA axis and the oxytocinergic system. CRH and oxytocin produced in the PVN exerts counterbalancing effects; CRH and oxytocin fibres originating in the PVN run in parallel to important stress regulatory centres allowing competition between these two substances; PVN neurons that project to the median eminence produce and release CRH in response to stress, increasing HPA-axis activity. Oxytocin, counterbalances this, exerting strong inhibitory effects on the HPA–axis in three different ways: decreasing the secretion of CRH from neurons in the PVN; inhibiting ACTH secretion from the anterior pituitary; and furthermore, oxytocin may decrease the release of cortisol by a direct mechanism in the adrenals. Oxytocin inhibits the secretion of ACTH in two ways, via axon collaterals projecting into the median eminence from the magnocellular neurons from the SON and PVN, which project to the posterior pituitary as well as by a bundle of parvocellular oxytocinergic neurons from the PVN projecting into the median eminence. Oxytocin released into the median eminence then reaches the ACTH-producing cells in the anterior pituitary, where they inhibit ACTH secretion and consequently the levels of cortisol. Oxytocin actions and fibres in brown; CRH, ACTH actions and fibres in blue.

The most well-known effects of oxytocin are stimulation of uterine contractions during birth and milk ejection in connection with breastfeeding. In these situations oxytocin is released from the anterior pituitary into the circulation and thus acts as a hormone. Oxytocin may, however, also be released as a neurotransmitter in the brain (**Figure 2A**). In the present paper we will focus on the neurotransmitter effects of oxytocin and in particular those involved in inhibition of stress levels. The oxytocin released into the circulation and the brain constitute separate compartments, as oxytocin does not pass the blood brain barrier, due to its chemical properties. Only 1 permille of a dose of oxytocin given peripherally enters the brain (82). Still there are connections between the periphery and the brain, as one of the major releasers of oxytocin is mediated via sensory nerves in particular via parasympathetic sensory nerves and cutaneous afferents. Some studies suggest that oxytocin may be transported from the circulation to the amygdala via a specific transport system, the receptor for advanced glycation end-products (RAGE) (83).

Oxytocin acts via activation of specific receptors of the G-receptor type. OTRs are located in most peripheral tissues as well as in the central nervous system. Until now only one type of OTR has been identified, but OTR (sequence) variants play a significant role in determining the cellular location of the OTR. Two variants (E339K and V281M) result in the receptor never reaching the outer membrane, rather being sequestrated intracellularly.

### 3.1 The tissue distribution of the oxytocin system

The nonapeptide oxytocin is produced by magnocellular neurons from the supraoptic (SON) and paraventricular (PVN) nuclei of the hypothalamus. These neurons of both these nuclei project to the posterior pituitary. Here, neurophysin-bound oxytocin is stored in Herring bodies at the axon terminals from where it is released into the circulation, e.g. during birth and breastfeeding. At the same time, local oxytocin release from magnocellular neuron dendrites and cell bodies exerts central (cerebral) effects (84, 85). Oxytocin release is subjected to a positive forward circle, i.e. that oxytocin released at the dendritic level further stimulates oxytocin release.

In addition, parvocellular neurons of the PVN produce and secrete oxytocin. These parvocellular neurons can be subdivided into several separate bundles that project to tightly delineated regulatory areas. One group of oxytocinergic nerves terminates in the median eminence allowing further transport of oxytocin into anterior pituitary. Other groups of nerves project to the LC, the rostroventrolateral medulla (RVLM), the sympathetic ganglia, the NTS and the dorsal motor nucleus of the vagus (DMX) and the amygdala. Oxytocin fibres also project to the periaqueductal grey (PAG). Other fibres reach the spinal cord and connect with parasympathetic centres in the lumbosacral region as well as afferent fibres mediating pain in the dorsal column. Other areas within the brain that are reached by the oxytocin neurons are the amygdala, hippocampus, raphe nuclei (RN), striatum, NA as well as numerous cortical areas (86–88). In addition, to the oxytocinergic fibres that emanate from the PVN, extrahypothalamic areas in the brain may be influenced via axon collaterals from the magnocellular neurons emanating in the SON and PVN, which project to the posterior pituitary. Such axon collaterals have been shown to project to the median eminence, hippocampus, the amygdala, and various cortical areas (89–92) (**Figure 2B**). Oxytocin is also released into the cerebrospinal fluid via nervous projections from the PVN allowing effects in other parts of the brain. CSF levels of oxytocin exhibit a diurnal rhythm, which is not the case for circulating oxytocin levels, again suggesting a differential regulation of the two compartments (93).

### 3.2 Anti-stress effects of oxytocin

One facet of oxytocin is, after low-medium intensity stimulation, its role as the antithesis of cortisol and the stress system. However, after intense (pain) stimuli this may not be the case, and stress may be activated. It exerts a plethora of anti-stress effects including: decreasing cortisol levels and dampening the SNS; pain relief; stimulation of bonding and social interaction; as well as both promoting growth and having restorative effects (94, 95). Oxytocin may exert physiological and psychological direct effects via activation of OTRs, but often acts indirectly by promoting or inhibiting the release of other neurotransmitters or by influencing the activity of the sympathetic or parasympathetic branches of the autonomic nervous system. For example, oxytocin exerts pain-relieving effects in the periaqueductal grey (PAG) via activation of opioidergic mechanisms and in part decreases stress levels by activation of alpha 2 adrenoceptors. By activation of the dopaminergic and the serotonergic neurons in the NA, striatum and RN oxytocin is involved in the control of mood, well-being and bonding (86–92). By projecting to the amygdala, BNST and hippocampus and several areas within the cortex, oxytocin influences stress levels, but may also promote different aspects of social interaction and participate in emotional experiences (89–92, 94, 96–100). These effects will not be further discussed in this paper. Below, we will summarize the different anti-stress effects of oxytocin taking place at the level of the PVN, the anterior pituitary, the presynaptic sympathetic ganglia, DMC and NTS, the LC and RVLM, as well as the amygdala, BNST and the hippocampus.

### 3.3 Sensory stimulation induces the release of oxytocin

Oxytocin release is under multifactorial control. Hormones such as oestrogen may stimulate oxytocin release and the number and function of OTRs in some areas. Also some types of stress are linked to oxytocin release, and in these situations oxytocin often serves to modify or buffer the stress responses. However, there are stimuli, of both central and peripheral origin which exclusively activate those aspects of the oxytocin system, which are linked to calm, stress reduction and promotion of healing and growth. One of the most important stimuli for this kind of oxytocin release is afferent sensory stimulation of low intensity, e.g. via afferent parasympathetic/vagal pathways but also via cutaneous afferent nerves.

### 3.4 Sensory nerves mediating the release of oxytocin

During parturition and the immediate post-partum period as well as during breastfeeding different types of sensory nerves are activated, which stimulate oxytocin release; parasympathetic sensory nerves from the cervix and the vagina in connection with birth, cutaneous sensory nerves from the nipple during suckling and cutaneous sensory afferents as well as afferent vagal nerves originating in the skin of the chest during skin to skin contact.

Afferent, sensory stimulation from the cardiovascular, gastrointestinal and urogenital tract and a subpopulation of skin afferents from the chest are mediated via parasympathetic/vagal afferents which terminate in the nucleus tractus solitarius (NTS), which is the primary relay centre for information from the autonomic nervous tone. The NTS is connected with the catecholaminergic, “noradrenergic” A2 neurons in the brain stem, which in turn project directly to the oxytocin neurons in the SON and PVN. Both magnocellular and parvocellular neurons can be activated in this way.

Afferent sensory nerves from the skin can also induce oxytocin release. Both unmyelinated C and myelinated A-fibres which respond to mechanical stimulation and temperature of the skin are involved. Most myelinated afferent fibres enter the ipsilateral dorsal column-medial lemniscus tract (posterior column pathway) and later cross to the contralateral spinothalamic pathway. On the other hand, both unmyelinated and thinly myelinated afferent fibres run in the contralateral spinothalamic tract to supraspinal levels (spinothalamic pathway). While travelling to the thalamus and cortex, the sensory fibres send axon collaterals to the oxytocin producing neurons in the SON and PVN where they may activate oxytocin synthesis/release. The cutaneous afferents also connect with the NTS, which in turn is connected with the SON and PVN. Some cutaneous nerves from the mammary gland project to the ganglion nodosum and the NTS, thus bypassing the spinal cord. In addition, some of the parasympathetic afferents projecting into the NTS from the urogenital tract run in front of the spinal cord (101) (**Figure 2B**).

### 3.5 Somatosensory nerve stimulation increases oxytocin release/production

In support of a connection between cutaneous sensory nerves and the oxytocin system, stimulation of sensory nerves originating in the skin induces oxytocin release into the (peripheral) circulation as well as in the brain. Low-intensity electrical stimulation of the sciatic nerve in rats increased plasma oxytocin levels in a direct intensity-related manner. Furthermore, tactile stimulation such as the application of warmth or massage to the abdominal/ventral area, or the brushing of a leg increased oxytocin both circulating and cerebral oxytocin levels (102–104). Furthermore, somatosensory stimulation does not simply modulate or initiate the release of available oxytocin reserves but increases the levels of oxytocin mRNA in both the PVN and SON (105).

### 3.6 Stress and oxytocin release

Oxytocin release is not limited to non-noxious (i.e. innocuous) sensory stimulation as noxious somatosensory stimuli such as various stressors can also induce its synthesis and release from the SON and/or PVN (102). Here, oxytocin either buffers the stress response or exerts other protective effects. When a stressor becomes repetitive there is a gradual decrease in CRH production that will eventually be extinguished as the concomitant oxytocin levels gradually rise. As such, oxytocin would appear to play a central role in stress habituation (105, 106).

### 3.7 Noxious and non-noxious stimulation of somatosensory nerves induce opposite physiological effect patterns

Innocuous and noxious stimulation of cutaneous afferents do, however, produce opposite effect patterns. As well as inducing fear and pain, noxious stimulation increases heart rate and blood pressure as well as the stress hormones cortisol, adrenaline, and noradrenalin. Furthermore there is evidence that it also negatively influences the level of gastrointestinal hormones and gastrointestinal function. Innocuous stimulation, on the other hand, decreases cortisol, noradrenalin and adrenaline levels and reduces both heart rate and blood pressure. Gastrointestinal-function is also enhanced while the pain threshold is lowered and sedation is induced after innocuous stimulation. This can be summarized as the activation of the HPA-axis and of the SNS, and a negative impact on vagal nerve activity and G-I tract function of noxious stimulation (107). In contrast, HPA-axis activity is decreased as is the SNS function whereas the activity of the PNS is enhanced after innocuous stimulation (94, 98). Stimulation of the cutaneous nerves induces these physiological effects via axon collaterals to the PAG and the NTS, which activate hypothalamic CRH and oxytocin producing neurons in response to noxious and innocuous stimulation respectively. Interactions between SNS and PNS may also occur at the level of the NTS.

## 4 Oxytocin plays an important role in regulating the stress response

As mentioned above, CRH produced in parvocellular neurons of the PVN exerts an important role for the activation of stress reactions mediated via the HPA axis and the SNS arm of the autonomic nervous system. In contrast, oxytocin which is also produced in parvocellular neurons of the PVN, exerts potent stress inhibitory effects on these functions under most conditions, although intense (pain) stimuli may have the opposite effect. In many cases CRH and oxytocin fibres originating in the PVN run in parallel to important stress regulatory centres allowing competition between these two substances (**Figure 2B**).

### 4.1 Oxytocin regulates the HPA - axis

PVN neurons that project to the median eminence produce and release CRH in response to stress, increasing HPA-axis activity. As outlined above, CRH is then transported to the anterior pituitary, where it induces ACTH release, which in turn stimulates the adrenal cortex to release cortisol. Oxytocin, counterbalances this, exerting strong inhibitory effects on the HPA–axis in three different ways: decreasing the secretion of CRH from neurons in the PVN; inhibiting ACTH secretion from the anterior pituitary; and furthermore, oxytocin may decrease the release of cortisol by a direct mechanism in the adrenals.

Oxytocinergic neurons within the SON and PVN project to GABAergic interneurons located outside the PVN. These GABAergic neurons then provide negative feedback, projecting back to the PVN and inhibiting CRH secretion in a GABA A receptor-mediated process (105, 108–110). Oxytocin inhibits the secretion of ACTH in two ways, via axon collaterals projecting into the median eminence from the magnocellular neurons from the SON and PVN, which project to the posterior pituitary as well as by a bundle of parvocellular oxytocinergic neurons from the PVN projecting into the median eminence (**Figure 2B**). Oxytocin released into the median eminence then reaches the ACTH-producing cells in the anterior pituitary, where they inhibit ACTH secretion and consequently the levels of cortisol. Oxytocin may also directly inhibit the release of cortisol by actions in the adrenal cortex (111).

### 4.2 Oxytocinergic and CRH neurons regulate the parasympathetic and sympathetic nervous systems

CRH neurons from the PVN project to many centres regulating autonomic tone such as the RVLM, LC, NTX, DMX and the preganglionic sympathetic ganglia. In these centres, CRH simultaneously upregulates sympathetic nerve activity (including the SAM) while inhibiting parasympathetic nervous activity (25, 112). Oxytocinergic neurons project from the PVN parallel to the CRH neurons to exactly the same centres regulating autonomic tone inducing an opposite reaction, inhibiting sympathetic nerve activity while stimulating parasympathetic nerve activity (94, 98).

This parallel organization of oxytocinergic and the CRH neurons, together with the fact that oxytocin and CRH exert opposite actions on the stress system provides a solid anatomical basis for a “competition”, or “counterbalancing” action between the two systems regulating HPA – axis function and sympathetic and parasympathetic nervous system activity. The higher the activity in the CRH neurons, the lower the oxytocin linked anti-stress effects and the higher the activity in the oxytocinergic neurons the lower the CRH linked stress.

### 4.3 The locus coeruleus – mediating the two systems

The LC is one of the most important centres for stress regulation. One of the most important pathways by which the LC is activated originates in the amygdala, which in turn receives information regarding fear from the prefrontal cortex (PFC). CRH neurons originating in the amygdala and which project to the LC, activate noradrenergic neurons in response to stress. Noradrenergic neurons from the LC project into the PVN, increase CRH neuron activity and consequently increase HPA axis and the sympathetic nervous system activity (25). Furthermore, noradrenergic neurons from the LC project directly to the autonomic nervous centres, where they increase the activity of the sympathetic nervous systems (110). CRH neurons from the PVN also promote NA release in the LC (25). As such, the two most important stress regulatory centres in the brain, the CRH neurons in the PVN of the hypothalamus and noradrenergic neurons from the LC in the brainstem mutually act synergistically, mutually reinforcing their function and modulating stress levels (25, 110).

The LC receives oxytocinergic signals from nerve fibres originating in the PVN. These have a strong anti-stress effect, decreasing LC activity through a direct inhibition of NA neuron firing (113). This direct effect on the noradrenergic neurons is supported indirectly by the oxytocin-mediated decrease of the activity of hypothalamic CRH mediated input to the LC. Since the secretion of CRH in the PVN is increased by noraderenergic neurons emanating from the LC, when LC noradrenergic activity is inhibited by oxytocin CRH secretion and synthesis will be decreased. Consequently, HPA-axis and SNS activity will be decreased and the counterbalancing parasympathetic nervous system activity will be increased (94, 98, 106, 112–114).

#### 4.3.1 Role of alpha 2-adrenoceptors

In addition to classical OTR mediated actions, the anti-stress effects of oxytocin also involve alpha 2 adrenoceptors, which inhibit noradrenergic function. Oxytocinergic activation of alpha 2 adrenoceptors is particularly prominent after long term exposure to oxytocin and is associated with long lasting effects. When oxytocin is administered repeatedly sensitivity of single cell-NA neurons in the LC to clonidine, an alpha 2-adrenoreceptor agonist, increases markedly, as measured by single cell recordings. At the same time, repeated exposure to oxytocin increases both the density and effectiveness of alpha 2-adrenoreceptors in all areas involved in autonomous nervous control (113, 114). Reduced blood pressure after repeated oxytocin administration is counteracted by alpha 2 antagonists, demonstrating that the effect was induced by an increased alpha 2 adrenoceptor function. Also vagal nerve function is increased in response to such stimulation. When oxytocin is administered for 5 days after birth to newborn rat pups, a lifelong increase in the number/function of alpha 2-adrenoreceptors are obtained. In addition to a lifelong lowering of cortisol levels and blood pressure is induced by this treatment. Taken together, these data suggest that exposure to oxytocin release in connection with birth and in the perinatal period might influence the function of the stress axis via an increase of the activity of the alpha 2 receptor system over the long-term.

### 4.4 The amygdala – social interactions and dampening stress

The amygdala is an important centre for fear and social interaction regulation. As mentioned above, external danger signals activate specific CRH neurons projecting from the central nucleus of the amygdala (CEA) to the LC, consequently increasing NA neuron activity (21, 25). As a consequence, the activity of both the HPA axis and the SNS will be increased. The CEA also receives input from oxytocinergic nerves emanating from the PVN as well as axon collaterals from oxytocinergic neurons from the magnocellular neurons from the SON (89–92). Consequently, oxytocin decreases the levels of CRH in the CEA via a GABAergic mechanism (115). This means that, oxytocin, for example when it is released in response to somatosensory stimulation, will not only decrease the amygdala-dependent fear sensations, but also the activity of the CRH neurons which originate in the CEA and project to the LC. In this way oxytocin indirectly contributes to decreased activity of both the HPA-axis and the SNS. Oxytocin fibres in the hippocampus and in the BNST may further contribute to the anti-stress effects of oxytocin. However, there is also evidence that the opposite may occur, where OT infusion into anteromedial BNST mice that have not been exposed to stress had reduced social approach and increased vigilance suggesting a role in anxiety-related behaviour (116).

In summary, oxytocin is an extremely powerful anti-stress hormone. These anti-stress effects depend on oxytocinergic nerves emanating from the SON and PVN, and project to multiple sites at which the stress system is regulated. These, include projections to CRH neurons within the PVN, to the anterior pituitary, to areas involved in sympathetic and parasympathetic nervous control, to NA neurons in the LC, and to CRH neurons in the amygdala. In all of these regions and nuclei oxytocin may be released after e.g. stimulation of cutaneous nerves. The importance and relevance of this, particularly in the early-life period is further examined below.

## 5 Immediate and long-term effects of oxytocin during labour, birth, skin-to-skin postpartum contact and breastfeeding

In the context of the interaction between the HPA axis and the oxytocin system birth is a particularly interesting period as, for both the mother and the infant, both systems are very strongly activated within the same narrow time window. During labour and birth, both the stress system and the oxytocin system are activated in both the mother and foetus. Moderate pressure in the cervix caused by the head of the foetus will activate parasympathetic afferents which stimulates oxytocin release and the parasympathetic nervous system. On the other hand, strong uterine contractions leading to hypoxia and metabolic changes in the uterine muscles in connection with birth will trigger the activity of sympathetic afferent nerves from the uterus which may consequently induce pain and fear and activation of the maternal stress system. Analogous physical stimuli may influence the stress and anti-stress systems of the foetus during birth. We can learn many lessons on the interaction between the two systems from this period.

### 5.1 Childbirth – intense activation of oxytocin and stress systems in both mother and baby

Maternal HPA axis activity and both maternal and foetal cortisol levels rise throughout pregnancy, reaching their zenith during birth (117–119). This rise plays an essential role in the terminal development of foetal lungs and initiating pulmonary surfactant production (120), as well as controlling hepatic (121), cardiac (122), cardiovascular (123), and G-I tract (124) maturation, as a coordinated series of events preparing the foetus for the transition to extra-uterine life. Until late in gestation, foetal cortisol is principally of maternal origin despite the presence of protective placental enzymes such as 11β HSD (125). However, it has also become clear that while maternal HPA axis set-points are naturally increased during pregnancy, further elevation due to stress brings negative consequences, particularly for the baby (126, 127), while maintaining them at pre-pregnancy levels also has considerable negative consequences, increasing the risk of premature labour as well as neonatal morbidity (128). Postpartum, the maternal HPA axis returns to the pre-partum state over a period of around 12 weeks, with CRH secretion from the hypothalamus reduced up to six weeks postpartum. The infant, however, enters into a stress hypo-responsive period (SHRP; reviewed in (129)). During this period, the infant’s HPA axis is, in particular levels of glucocorticoids from the adrenal gland, are significantly less sensitive to external stressors. Here, pituitary feedback mechanisms appear to downregulate HPA axis activity, although remain functional and capable of inducing a suitable, albeit reduced response to stressors present in the environment.

In a similar manner, oxytocin levels increase between 3- and 4-fold during pregnancy. The increase in both amplitude and frequency of oxytocin pulses rapidly increase oxytocin levels during the first two stages of labour (reviewed in (130). Oxytocin released during labour has two principal effects; circulating oxytocin stimulates the uterus to contract, while localised release in specific cerebral loci influences maternal physiology and behaviour during birth. The anti-stress effects of oxytocin are clearly seen during birth and immediately post-partum. Naturally released oxytocin during vaginal birth has been demonstrated to alter social interactions, increase the feeling of well-being as well as reducing anxiety and pain (130). Oxytocin release, mediated by skin-to-skin contact immediately post-partum, has a major stress-reducing effect. Anxiety levels are reduced in both the mother and infant and at the same time, cortisol levels rapidly diminish. This is accompanied by pulse rate regulation in the newborn as well as facilitation of the mother-infant interaction (131).

### 5.2 Breastfeeding and oxytocin release

The rapid pulses of oxytocin observed in the circulation during breastfeeding are associated with contraction of the myoepithelial cells in the mammary glands, in order to promote milk ejection. Furthermore, the psychological and physiological effects of breastfeeding are mediated by the central oxytocinergic system (132). There is a marked decrease in blood pressure as well of HPA axis mediators and effectors (ACTH, cortisol) in response to each suckling episode. At the same time oxytocin influences the DMX and via activation of the vagal nerve maximizes digestive capacity and anabolic processes (133). Oxytocinergic nerves projecting to the amygdala and other regions involved in social interaction and regulation of fear and pain, stimulates social interaction and decreases fear pain. Furthermore, nerves projecting to the dopamine neurons in the reward centre leads to increased feelings of wellbeing (134).

The anti-stress effects caused by breastfeeding may be turned into long lasting effect. After 6 weeks of breastfeeding basal blood pressure is significantly decreased, indicating a more long term anti-stress effect in response to repetitive breastfeeding (135). Despite this decrease of basal levels of blood pressure each breastfeeding episode is linked to a short term decrease of blood pressure. Also the activity of the HPA axis is down regulated by breastfeeding and physical exercise induces a reduced effect on the release of ACTH and cortisol in breastfeeding women when compared to non-breastfeeding women (136).

There is an interesting difference between the stress reduction in response to suckling/breastfeeding and skin-to-skin treatment. Suckling induces a decrease of ACTH levels which parallels that of the oxytocin release into the circulation. This effect is most likely induced by oxytocin released into the median eminence form the axon collaterals of the oxytocin nerves projecting to the posterior pituitary (see above).

In contrast skin to skin gives rise to decreased cortisol levels in the absence of both rising oxytocin levels and decreasing ACTH levels. We have interpreted this phenomenon as follows: The cutaneous afferents which are activated by skin-to- skin contact do not activate the magnocellular oxytocin neurons to the same extent as does suckling, which is a more intense stimulation. Instead the parvocellular aspect of the oxytocin system may be activated and thereby the activity of the sympathetic nervous system is decreased leading to a different sensitivity of the ACTH receptors in the adrenal cortex (135).

Exactly the same twofold types of regulation of cortisol release have been demonstrated in lactating cows. The milking machine induces a rise of oxytocin levels and a decrease of ACTH levels and of cortisol levels. By contrast stroking of the ventral area in front of the udder, is followed by a decrease of cortisol levels but not of ACTH (137). These differences between suckling and touch induced reduction of cortisol levels suggest a different arrangement of the oxytocin linked stress reducing mechanisms. The suckling induced effect involves the magnocellular neurons and the HPA axis in a classical sense, whereas skin to skin contact activates the parvocellular aspect of the oxytocin system which induces anti stress effects by reducing the activity of the sympathetic input to the adrenal glands. It is likely that the skin which is a less specialized organ than the mammary gland and the uterus has retained a more primitive organization regarding its afferent sensory mechanisms that influences CNS function. Also the newborn who is suckling activates its oxytocin system through pressure and touch of the oral mucosa. In this way stress levels are reduced, calm is induced (pacifier) and also metabolic processes, that optimize growth (133).

It should be noted that skin-to-skin is not only effective with maternal contact, but there is also a bidirectional paternal effect with both paternal oxytocin levels increasing (138) and infant stress and cortisol levels being lowered (139).

### 5.3 The SHRP or the stress hyporesponsive period

Translating from rodent to human experiments is made particularly complicated because of the stress hyporesponsive period (SHRP). This is a term derived from rodent experiments in which a lower sensitivity to stress has been shown during the post-natal period. Although this term is rarely used in the human literature, there is (limited) human evidence that the SHRP exists in infancy, potentially extending into early childhood (140, 141). Unlike rodent models, new-born (human) infants can produce an efficient HPA axis stress response, although the sensitivity of the reaction diminishes over the first 12 months of life, and is dependent on both social stimulation and parental care (140, 141). It has also been suggested that absence of either (or both) of these elements disrupts both the SHRP and the subsequent development of the HPA axis response to a stressor (21, 142) in humans. It is clear, however, from the rodent literature that oxytocin continues to be released throughout the SHRP. Many sensory stimuli received during (although not unique to) this period induce specific patterns of oxytocin release. Breastfeeding is, for example, associated with rapid short, sharp “peak-shaped” pulses of circulating oxytocin peripherally, whereas mother-infant skin-to-skin contact induces slower oxytocin pulses that are less frequent, less intense, but last longer (95, 130, 132, 143).

### 5.4 Mechanisms linking oxytocin and the SHRP

There is a clear link between oxytocin release and stress hyporesponsivity in the SHRP, however, the mechanism isn’t clear-cut (reviewed in (144)). While it is clear that intracerebroventricular infusion of oxytocin almost completely abrogates the neuroendocrine and SNS response to a stressor (145) and intracerebroventricular oxytocin antagonist infusion increased basal and post-stress corticosterone levels (146) this effect hold true for healthy female adult rats, but is lost in late pregnancy and lactation (146). When we examine the SHRP in more detail, in many circumstances, the hypothalamus remains reactive with increased Fos expression as well as increase circulating levels of ACTH, corticosterone/cortisol, oxytocin as well as AVP (reviewed in (147)). This may, in part be due to the immaturity of the ascending viscerosensory pathways to oxytocinergic centres at birth and during the SHRP.

While there are clear neuronal mechanisms in play, there are also long term effects beyond the SHRP that appear to be put in place during this time. In a manner similar to the adverse environment-induced epigenetic regulation of the HPA axis reactivity, the NR3C1 gene is also epigenetically regulated by positive environmental influences (reviewed in (148)). However, the sparse literature available suggests that breastfeeding and more general “maternal care” decrease DNA methylation, reducing NR3C1 expression and HPA axis reactivity to social stressors later in life (149, 150), in many ways mirroring the original data of Weaver et al. that initiated the field of behavioural epigenetics where low caring rat dams induced high Nr3c1 methylation levels and high caring dams induced low methylation levels in their pups (71). Furthermore, sub-optimal maternal touch had the opposite effect, increasing methylation levels, especially in girls (151). It is clear that the stress and anti-stress systems are intertwined, because, at the same time, the OTR gene (gene symbol *OXTR*) is concurrently differentially methylated by maternal care (152). Increased or high quality maternal care decreased *OXTR* promoter methylation (152), and levels of maternal touch were predictive of reduced *OXTR* methylation levels up to 18 months later (153).

Taken together, these data suggest that the HPA axis and the oxytocin system appear to be both regulated in this early life period. Furthermore, external signals regarding fear and safety will add to the activity in the stress and oxytocin system respectively. As somatosensory inputs appear to epigenetically regulate the HPA axis as well as the oxytocin system this of course means that it is of utmost importance to stimulate the oxytocinergic system as much as possible in connection with labour and birth, maintaining stress levels under control and avoiding long term complications associated with pathologically elevated stress/cortisol levels. Such stimulation may be induced by creating a safe environment, by support from a helping person or even by touch or massage. This is not only of importance for the progress and experience of labour it may also have some positive effects in the long term since it may reduce the risk for the development of postpartum anxiety disorders. From this perspective, the practice of skin-to-skin after birth is important, since it is connected with the activation of the stress-reduction facet of the oxytocin system. This will of course further reduce the possibility of negative stress-related consequences after birth.

## 6 Conclusion

**Figure 3:**
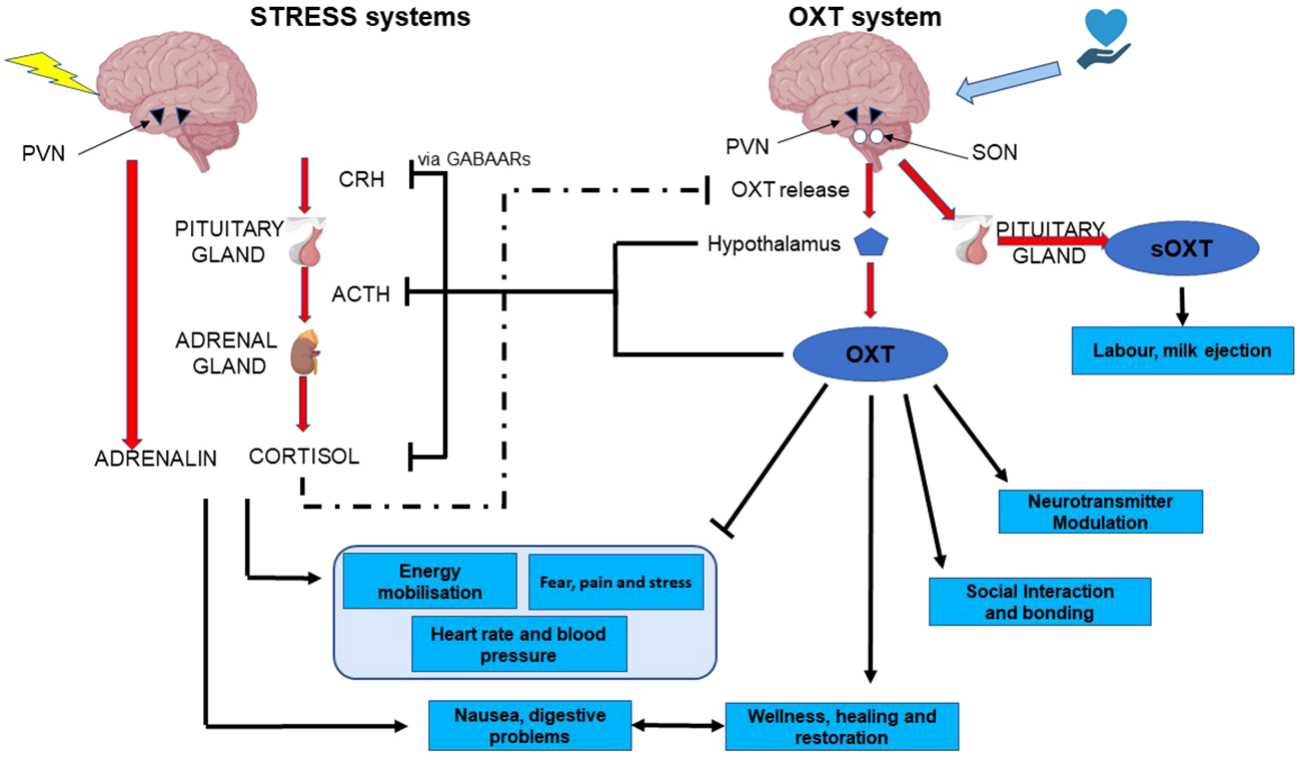
functional and mechanistic interactions between the two arms of the stress system and the two arms of the oxytocin system. CRH, corticotropin-releasing hormone; ACTH, adreno-corticotropic hormone; PVN, pDaraventricular nucleus of the hypothalamus; OXT, oxytocin; sOXT, secreted oxytocin; SON, supraoptic nucleus of the hypothalamus.

Throughout this review we have emphasised the opposite, yet interdependent and intertwined nature of the oxytocin and stress systems. Although the HPA axis and glucocorticoid stress axis are both well studied, the extremely powerful anti-stress facet of oxytocin is somewhat underappreciated. It is clear that these anti-stress effects depend on oxytocinergic nerves emanating from the SON and PVN, and project to multiple sites at which the stress system is regulated (**Figure 3**). These, include projections to CRH neurons within the PVN, to the anterior pituitary, to areas involved in sympathetic and parasympathetic nervous control, to NA neurons in the LC, and to CRH neurons in the amygdala. In all of these regions and nuclei, oxytocin may be released after activation of cutaneous nerves in response to low intensity stimulation. However, there is now evidence that the two systems come into play in the SHRP, and that it may be somatosensory inputs, acting through the oxytocinergic system that underlie this period of hyporeactivity. Furthermore, this is a period in which, despite its hyporeactivity, the stress system is epigenetically programmed by its environment, potentially by oxytocinergic inputs, and vice versa.

Experienced midwives and clinicians are perhaps aware of the balances or possible imbalances between the stress and oxytocin systems which are involved while a mother is giving birth. Regular contractions combined with deep vocalization, dancing movements, sighs and exhaustion may be promising signs for a balanced oxytocin and stress system which is also affecting the well-being of the unborn child. Oxytocin peaks during birth lead to deep sensations which may be remembered by the mother throughout life while the newborn is being nurtured with oxytocin and endorphins. During childbirth and breastfeeding, there can be physiological and psychological stress which can have negative effects on the delivery, lactation and bonding. Oxytocin plays a key role in regulating processes that ensure both the maternal and child health in the peripartum period. Oxytocin always needs to be regarded as promoting the return to homeostasis and starting the post-stress healing process. Oxytocin and the stress pathways behave as the Yin Yang systems, which control each other on a macro and microlevel. However, as we have highlighted, the two systems have been investigated largely independently. We suggest that these two systems are very closely intertwined, and represent two sides of the same coin, balanced as Yin and Yang. Only by taking the two systems together will we be fully able to determine their effects on birth outcomes, maternal health, and infant well-being.

## Author contributions

KU-M: Conceptualization, Formal analysis, Writing – original draft, Writing – review & editing. MG: Writing – original draft, Writing – review & editing. JC-A: Writing – original draft, Writing – review & editing. JT: Conceptualization, Formal analysis, Writing – original draft, Writing – review & editing.
